# Causes of Death among Adults in Northern Ethiopia: Evidence from Verbal Autopsy Data in Health and Demographic Surveillance System

**DOI:** 10.1371/journal.pone.0106781

**Published:** 2014-09-04

**Authors:** Yohannes Adama Melaku, Berhe Weldearegawi Sahle, Fisaha Haile Tesfay, Afework Mulugeta Bezabih, Alemseged Aregay, Semaw Ferede Abera, Loko Abreha, Gordon Alexander Zello

**Affiliations:** 1 Department of Public Health, College of Health Sciences, Mekelle University, Mekelle, Ethiopia; 2 School of Medicine, College of Health Sciences, Mekelle University, Mekelle, Ethiopia; 3 College of Pharmacy and Nutrition, University of Saskatchewan, Saskatoon, Canada; Columbia University, United States of America

## Abstract

**Background:**

In countries where registration of vital events is lacking and the proportion of people who die at home without medical care is high, verbal autopsy is used to determine and estimate causes of death.

**Methods:**

We conducted 723 verbal autopsy interviews of adult (15 years of age and above) deaths from September 2009 to January 2013. Trained physicians interpreted the collected verbal autopsy data, and assigned causes of death according to the international classification of diseases (ICD-10). We did analysis of specific as well as broad causes of death (i.e. non-communicable diseases, communicable diseases and external causes of death) by sex and age using Stata version 11.1. We performed logistic regression to identify socio-demographic predictors using odds ratio with 95% confidence interval and a p-value of 0.05.

**Findings:**

Tuberculosis, cerebrovascular diseases and accidental falls were leading specific causes of death accounting for 15.9%, 7.3% and 3.9% of all deaths. Two hundred sixty three (36.4% [95% CI: 32.9, 39.9]), 252 (34.9% [95% CI: 31.4, 38.4]) and 89 (12.3% [95% CI: 10.1, 14.9]) deaths were due to non-communicable, communicable diseases, and external causes, respectively. Females had 1.5 times (AOR = 1.53 [95% CI: 1.10, 2.15]) higher odds of dying due to communicable diseases than males. The odds of dying due to external causes were 4 times higher among 15–49 years of age (AOR = 4.02 [95% CI: 2.25, 7.18]) compared to older ages. Males also had 1.7 times (AOR = 1.70 [95% CI: 1.01, 2.85]) higher odds of dying due to external causes than females.

**Conclusion:**

Tuberculosis, cerebrovascular diseases and accidental falls were the top three causes of death among adults. Efforts to prevent tuberculosis and cerebrovascular diseases related deaths should be improved and safety efforts to reduce accidents should also receive attention.

## Background

In most developing countries, where 80% of global deaths occur, registration of vital events is usually not carried out. The determination of causes of death in developing countries is difficult, as an overwhelming majority of deaths are neither attended by health professionals nor medically certified [1], [2], [3], [4], [5]. As a result, more than three quarters of the world’s population is not covered by routine registration of vital events [5].

Ethiopia is one of the countries without a vital registration system. Additionally, health service utilization is very poor with total outpatient use of government health facilities estimated at 0.25 visits per person per year [6]. Thus, due to poor access to health services and low healthcare seeking behavior, most deaths occur outside of health facilities. As a result, mortality data at both health facilities and in communities are lacking.

A death certificate completed by a physician with substantial knowledge of the clinical course of an individual prior to death based on appropriate diagnostics is the best standard for assigning cause of death. In countries where registration of vital events is incomplete(inaccurate) and the proportion of people who die at home without medical care is high, verbal autopsy (VA) is used to identify causes of death [3], [4], [5], [7], [8], [9] and measure patterns of causes of death [10]. Several studies showed that VA can be used to generate reasonable population level estimates of causes of death [11], [12]. Thus, the VA method is recommended to obtain a population level estimate of causes of deaths in the absence of a medical recording system [4], [13].

In recent years, concern has been raised on the inadequate attention in public health interventions and investment to prevent some health problems in adults [14], [15], [16] **.** A number of publications explored changing trends in all-causes and disease-specific mortality among adults in sub-Saharan Africa [17], [18], [19], [20], [21], [22]. While most global efforts to prevent mortality among young people focus in children below 5 years of age, significant health gains can also be attained among adults. However, target efforts for adults are suppressed by a lack of data [14], [23], [24].

The aim of this study was, therefore, to determine causes of death among adults (i.e. age greater than 15 years) in northern Ethiopia using data from the Kilite-Awlaelo Health and Demographic Surveillance System (KA-HDSS). Additionally, we explored the characteristics and trends (by sex and age) of broad causes of death (i.e. communicable diseases [CDs], non-communicable diseases [NCDs] and external Causes [ECs]).We also assessed the socio-demographic predictors of CDs, NCDs and ECs in an adult population.

## Methods

We have conducted the study among adults greater than 15 years of age and above (n = 723) from September 2009 to January 2013 as part of the ongoing KA-HDSS initiative. The KA-HDSS study site is located in a predominately rural part of the Tigray regional state in northern Ethiopia, specifically in the Kilte-Awlaelo, Wukro and Atsebe-Wonberta districts. The center is 835 kilometers north of Addis Ababa, the capital city of Ethiopia. The study area included 10 “Kebelles” (1 town and 9 rural areas) with a mid-year population of 65,120 living in 15,643 households in 2012/2013. In Ethiopia, Kebelles are smallest administrative units with an average population of 5,000–6,000. The population, mainly subsistence farmers, is almost exclusively members of “Tigrian” ethnic group. Overall, the population in this study site has lower mortality and fertility rates than other regions of Ethiopia as described elsewhere [25], [26].

### Verbal autopsy data

As part of KA-HDSS study, resident full-time enumerators, who visit each household every month for events, report all deaths in the surveillance site. Update rounds and update of the database are performed biannually. VA supervisors and trained VA data collectors reported deaths every month.

VA interviews were carried out between 45–55 days of the date of death in respect of the local mourning time. VA was conducted using a standardize World Health Organization (WHO) questionnaire endorsed by International Network for Demographic Evaluation of Populations and Their Health (INDEPTH) for all deaths occurring in the HDSS [27]. For this analysis, we utilized the adult questionnaire (15 years of age and above). We identified parents or spouses as the first respondents.

Reviewer physicians were trained on the application of the ICD-10 manual developed by WHO for assessing the global burden of diseases [28]. Two blinded physicians independently reviewed the completed VA questionnaires to assign cause of death using the manual. A Surveillance team member, who was in charge of this specific task, confirmed agreement between the two physicians. When disagreements in diagnosis arose, a third physician was assigned to review the case. The final diagnosis was assigned based on the agreement between the third physician and any of the two physicians. The case was considered as “undetermined” if all three physicians assigned a different diagnosis. Physician gave diagnosis a diagnosis “unspecified causes of death (VA-99)” for a case when difficulties to classify diseases based on the given information were present.

### Classification of causes of death

Based on the international disease classification system [28], [29] **,** we contextually categorized causes of death as follow:

#### Communicable diseases (CDs) (VA-01)

All infectious and parasitic diseases (VA-01) including Human immunodeficiency virus(HIV), tuberculosis, malaria, intestinal infection, infectious diseases of unspecified cause, acute lower respiratory infections, meningitis, viral hepatitis, typhoid and paratyphoid.

#### Non-communicable diseases (NCDs)

Diseases of circulatory system (VA-04), neoplasms (VA-02), renal disorders (VA-07), respiratory disorders (VA-05), gastrointestinal disorders (VA-06), mental and nervous system disorders (VA-08) and nutritional and endocrine disorders (VA-03). **External causes of death (ECs) (VA-11)**. Accidental falls, accidental drowning and submersion, intentional self-harm, assault and others which are not related to the above two categories.

#### Pregnancy, childbirth and puerperium related deaths (VA-09)

All deaths related to pregnancy and childbirths (e.g. maternal deaths associated with abortion, childbirth related hemorrhage).

### Data management and analysis

Analyses of data were performed with Stata version 11.1 (Stata Corporation, College Station, TX, USA) after exporting from the SPSS version 20.0 (IBM SPSS Statistics for Windows, Version 20.0. Armonk, NY: IBM Corp.). Frequencies, proportions and summary statistics were used to describe the socio-demographic characteristics of the deceased individuals. Differences in CDs, NCDs and ECs) of death, among groups were reported using Pearson’s chi-square (χ^2^) test.

Bivariate logistic regression was used to identify the crude relationship between the outcomes (CDs, NCDs and ECs) and the independent variables. The degree of association between outcome and some of the socio-demographic characteristics was assessed using odds ratio with 95% confidence interval. After testing for co-linearity [30] and interaction [31], all covariates with statistically significant associations in the bivariate analysis were retained in multivariate logistic regression model to obtain adjusted estimates of the association between covariates and outcome variables. All statistical tests were two-sided and considered statistically significant at a p-value of 0.05.

### Ethics statement

The KA-HDSS received ethical clearance from the Ethiopian Science and Technology Agency with identification number IERC-0030. Ethical approval was also obtained from the Health Research Ethics Review Committee (HRERC) of Mekelle University. Informed verbal consent was obtained from head of the family or an eligible adult in the family. This verbal consent was documented in English and local language “Tigrenga”. This documentation was done by marking “Yes” or “No” for a question “Are you willing to participate in this study?” after explaining all information about confidentiality, privacy and the right to not participate or withdraw from the study. VA data collectors continued the interviews after a study participant answered only “Yes”. The data collectors did this process for each of the study participant. All individuals who participated in this study had knowledge on verbal consent form. To keep confidentiality, data containing personal identifiers of subjects were not shared to any third parties. The above aforementioned institutions approved all these processes.

## Results

### Socio-demographic characteristics of the deceased

Of 723 documented deaths occurring during the study period in adults aged 15 years and above, an equal number were males and females (n = 361; 49.9%). Most of the deceased were from rural areas (n = 656; 91.1%). A quarter (n = 175; 24.2%) of all deaths occurred in the very old age group (75–84 years). The overall median age at death was 70 years (Inter Quartile range (IQR) = 50, 72 years). Approximately, four of five deaths (n = 578; 79.9%) occurred among illiterates and the majority deceased individuals were farmers (n = 328; 45.4%). Over half (n = 200; 55.4%) of deceased females were widowed and less than half (n = 313; 43.3%) of all individuals were married at time of death. Nine out of ten deaths (646; 89.4%) occurred out of health institutions **(** **Table 1** **)**.

**Table 1:** Socio-demographic characteristics of deceased adults in northern Ethiopia from September 2009 to January 2013 (n = 723).

		Male (n = 362)		Female (n = 361)		Total	
Variable	Category	n	%	n	%	n	%
**Residence**	Urban	27	7.5	37	10.3	64	8.9
	Rural	335	92.5	324	89.8	659	91.1
**Age at death (y)**	15–24	36	9.9	21	5.8	57	7.9
	25–34	24	6.6	29	8.0	53	7.3
	35–44	33	9.1	19	5.3	52	7.2
	45–54	18	5.0	32	8.9	50	6.9
	55–64	28	7.7	40	11.1	68	9.4
	65–74	69	19.1	65	18.0	134	18.5
	75–84	83	22.9	92	25.5	175	24.2
	85+	71	19.6	63	17.5	134	18.5
**Median(IQR)**		**70(43, 82)**		**70(53, 81)**		**70(50, 82)**	
**Education**	Illiterate	255	70.4	323	89.5	578	79.9
	Primary education	72	19.9	19	5.3	91	12.6
	Secondary education	27	7.5	16	4.4	43	5.9
	Higher education	8	2.2	3	0.8	11	1.5
**Occupation**	Farmer	236	65.2	92	25.5	328	45.4
	Student	21	5.8	39	10.8	60	8.3
	Private employee/merchant	6	1.7	9	2.5	15	2.1
	Shepherd	0	0.0	1	0.3	1	0.1
	Daily labor	22	6.1	12	3.3	34	4.7
	Housewife	0	0.0	72	19.9	72	10.0
	Government employee	9	2.5	7	1.9	16	2.2
	Unemployed	16	4.4	41	11.4	57	7.9
	Other	51	14.1	85	23.5	136	18.8
	Unknown	1	0.3	3	0.8	4	0.6
**Marital status**	Single	66	18.2	37	10.2	103	14.2
	Married	210	58.0	103	28.5	313	43.3
	Widowed	66	18.2	200	55.4	266	36.8
	Divorced	12	3.3	17	4.7	29	4.0
	Separated	1	0.3	0	0.0	1	0.1
	Unknown/missing	7	1.9	4	1.1	11	1.5
**Place of death**	Home	279	77.1	305	84.5	584	80.8
	Hospital	36	9.9	38	10.5	74	10.2
	Health center	3	0.8	0	0.0	3	0.4
	Other	44	12.2	18	5.0	62	8.6

### Specific causes of adult deaths

Of the specific causes of death, tuberculosis, cerebrovascular diseases and accidental falls were the leading causes accounting for 15.9%, 7.3% and 3.9% of all deaths, respectively. Nutritional and endocrine disorders, respiratory disorders and pregnancy related deaths were rare. Undetermined and unspecified causes of death were reported as 14.9% and 1.1% of all deaths, respectively **(** **Table 2** **)**.

**Table 2:** Specific causes of adult deaths by sex in northern Ethiopia from September 2009 to January 2013 (n = 723).

		Male (n = 362)		Female (n = 361)		Total		Rank
VA-title	VA-code	n	%	n	%	n	%	
**Infectious and parasite diseases**	**VA-01**	**105**	**29.0**	**147**	**40.7**	**252**	**34.9**	
Tuberculosis	VA-01.03	51	14.1	64	17.7	115	15.9	1
Intestinal infection diseases	VA-01.01	14	3.9	13	3.6	27	3.7	4
Infectious diseases, unspecified cause	VA-01.99	10	2.8	16	4.4	26	3.6	5
HIV/AIDS	VA-01.09	6	1.7	19	5.3	25	3.5	6
Acute lower respiratory infections	VA-01.13	10	2.8	14	3.9	24	3.3	7
Meningitis	VA-01.11	9	2.5	10	2.8	19	2.6	10
Malaria	VA-01.10	4	1.1	8	2.2	12	1.7	
Viral hepatitis	VA-01.08	0	0.0	2	0.6	2	0.3	
Typhoid and paratyphoid	VA-01.02	1	0.3	1	0.3	2	0.3	
**Diseases of the circulatory system**	**VA-04**	**50**	**13.8**	**50**	**13.9**	**100**	**13.8**	
Celebrovascular diseases	VA-04.03	27	7.5	26	7.2	53	7.3	2
Ischemic heart diseases	VA-04.02	8	2.2	12	3.3	20	2.8	9
Congestive heart failure	VA-04.05	11	3.0	5	1.4	16	2.2	
Hypertensive diseases	VA-04.01	4	1.1	6	1.7	10	1.4	
Chronic rheumatic heart disease	VA-04.04	0	0.0	1	0.3	1	0.1	
**External causes of death**	**VA-11**	**59**	**16.3**	**30**	**8.3**	**89**	**12.3**	
Accidental falls	VA-11.03	17	4.7	11	3.1	28	3.9	3
Accidental drowning and submersion	VA-11.04	12	3.3	3	0.8	15	2.1	
Intentional self-harm	VA-11.10	7	1.9	4	1.1	11	1.5	
Accident unspecified	VA-11.97	4	1.1	2	0.6	6	0.8	
Assault	VA-11.11	5	1.4	1	0.3	6	0.8	
Exposure to force of nature	VA-11.07	4	1.1	2	0.6	6	0.8	
Contact with animal venom	VA-11.06	2	0.6	3	0.8	5	0.7	
Pedestrian injured in traffic accident	VA-11.01	3	0.8	1	0.3	4	0.6	
Other transport accident	VA-11.02	3	0.8	1	0.3	4	0.6	
Accidental exposure to smoke, fire and flame	VA-11.05	0	0.0	1	0.3	1	0.1	
Lack of food	VA-11.09	0	0.0	1	0.3	1	0.1	
War	VA-11.13	1	0.3	0	0.0	1	0.1	
Other specified event, undetermined intent	VA-11.98	1	0.3	0	0.0	1	0.1	
**Neoplasm**	**VA-02**	**23**	**6.4**	**23**	**6.4**	**46**	**6.4**	
Malignant neoplasm of esophagus	VA-02.02	8	2.2	5	1.4	13	1.8	
Malignant neoplasm of small & large intestine	VA-02.04	5	1.4	2	0.6	7	1.0	
Malignant neoplasm of breast	VA-02.08	0	0.0	5	1.4	5	0.7	
Malignant neoplasm of hyphoid, hematopoietic and related tissue	VA-02.14	2	0.6	2	0.6	4	0.6	
Malignant neoplasm of cervix	VA-02.09	0	0.0	4	1.1	4	0.6	
Malignant neoplasm of stomach	VA-02.03	2	0.6	1	0.3	3	0.4	
Malignant neoplasm of rectum and anus	VA-02.05	0	0.0	2	0.6	2	0.3	
Malignant neoplasm of prostate	VA-02.12	2	0.6	0	0.0	2	0.3	
Neoplasm of uncertain or unknown behavior	VA-02.99	1	0.3	1	0.3	2	0.3	
Malignant neoplasm of trachea, bronchus and lung	VA-02-07	1	0.3	0	0.0	1	0.1	
Malignant neoplasm of uterus	VA-02.10	1	0.3	0	0.0	1	0.1	
Malignant neoplasm ovaries	VA-02.11	0	0.0	1	0.3	1	0.1	
Other specified neoplasm	VA-02.98	1	0.3	0	0.0	1	0.1	
**Mental and nervous system disorders**	**VA-08**	**23**	**6.4**	**13**	**3.6**	**36**	**5.0**	
Epilepsy	VA-08.02	14	3.9	8	2.2	22	3.0	8
Alzheimer disease	VA-08.01	6	1.7	3	0.8	9	1.2	
Specified disorders of the nervous system	VA-08.98	2	0.6	1	0.3	3	0.4	
Mental disorder unspecified	VA-08.99	0	0.0	1	0.3	1	0.1	
Other specified mental disorders	VA-08.96	1	0.3	0	0.0	1	0.1	
**Gastrointestinal disorders**	**VA-06**	**21**	**5.8**	**11**	**3**	**32**	**4.4**	
Chronic liver disease	VA-06.02	12	3.3	2	0.6	14	1.9	
Acute abdomen	VA-06.06	4	1.1	4	1.1	8	1.1	
Gastric and duodenal ulcer	VA-06.01	3	0.8	4	1.1	7	1.0	
Paralytic ileus and intestinal obstruction	VA-06-03	2	0.6	1	0.3	3	0.4	
**Renal disorders**	**VA-07**	**12**	**3.3**	**14**	**3.9**	**26**	**3.6**	
Renal failure	VA-07.01	11	3.0	14	3.9	25	3.5	6
Disorder of kidney & urether	VA-07.98	1	0.3	0	0.0	1	0.1	
**Nutritional and endocrine disorders**	**VA-03**	**13**	**3.6**	**3**	**0.8**	**16**	**2.2**	
Diabetes mellitus	VA-03.03	11	3.0	2	0.6	13	1.8	
Nutritional anemia	VA-03.01	2	0.6	0	0.0	2	0.3	
other specified endocrine disorder	VA-03.98	0	0.0	1	0.3	1	0.1	
**Respiratory disorders**	**VA-05**	**5**	**1.4**	**2**	**0.6**	**7**	**1.0**	
Asthma	VA-05.02	4	1.1	2	0.6	6	0.8	
Chronic obstructive lung disease	VA-05.01	1	0.3	0	0.0	1	0.1	
**Pregnancy, childbirth and postpartum**	**VA-09**	**0**	**0.0**	**3**	**0.8**	**3**	**3.9** *	
**Indeterminate**	**N/A**	**47**	**13.0**	**61**	**16.9**	**108**	**14.9**	
**Unspecified causes of death**	**VA-99**	**4**	**1.1**	**4**	**1.1**	**8**	**1.1**	

*-out of 77 reproductive age women (15–49 years), VA-Verbal Autopsy.

In the age group of 15–49 years, HIV/AIDS and tuberculosis were the leading causes of death accounting for 10.9% each. In the 50–64 years of age group, tuberculosis was the commonest cause of death at 27.2%. Cerebrovascular diseases were among the most frequent causes of death in the two age categories, 50–64 years and 65 years and above, at 7.8% and 10.1%, respectively. Causes of death were undetermined for 12.1%, 5.8% and 18.2% of deaths in age categories of 15–49 years, 50–64 years and 65 years and above, respectively **(** **Table 3** **)**.

**Table 3:** Specific causes of adult deaths by age in northern Ethiopia from September 2009 to January 2013 (n = 723).

Major causes of death by age group	VA-code	n	%	Rank
**15–49 years of age (n = 174)**				
**Infectious and parasitic diseases**	**VA-01**	**55**	**31.6**	
HIV/AIDS	VA-01.09	19	10.9	1
Tuberculosis	VA-01.03	19	10.9	1
**External causes of death**	**VA-11**	**50**	**28.7**	
Accidental drowning and submersion	VA-11.04	10	5.7	2
Intentional self-harm	VA-11.10	10	5.7	2
Accidental falls	VA-11.03	7	4.0	4
**Undetermined**	**Not applicable**	**21**	**12.1**	
**Mental and nervous system disorders**	**VA-08**	**10**	**5.8**	
Epilepsy	VA-08.02	9	5.2	3
**Gastrointestinal disorders**	**VA-06**	**9**	**5.2**	
Chronic liver disease	VA-06.02	6	3.4	5
**50–64 years of age (n = 103)**				
**Infectious and parasitic diseases**	**VA-01**	**39**	**37.9**	
Tuberculosis	VA-01.03	28	27.2	1
HIV/AIDS	VA-01.09	4	3.9	4
**Diseases of the circulatory system**	**VA-04**	**14**	**13.6**	
Celebrovascular disease	VA-04.03	8	7.8	2
**External causes of death**	**VA-11**	**11**	**10.7**	
**Neoplasm**	**VA-02**	**10**	**9.7**	
**Gastrointestinal disorder**	**VA-06**	**9**	**8.7**	
Chronic liver disease	VA-06.02	4	3.9	4
**Renal disorders**	**VA-07**	**6**	**5.8**	
Renal failure	VA-07.01	6	5.8	3
**Undetermined**	**Not applicable**	**6**	**5.8**	
**65 years of age and above (n = 446)**				
**Infectious and parasitic diseases**	**VA-01**	**158**	**35.4**	
Tuberculosis	VA-01.03	68	15.2	1
Intestinal infection disease	VA-01.01	24	5.4	3
Acute lower respiratory infections	VA-01.13	20	4.5	4
Infection, unspecified causes	VA-01.99	19	4.3	5
Meningitis	VA-01.11	15	3.4	
Malaria	VA-01.10	8	1.8	
**Undetermined**	**Not applicable**	**81**	**18.2**	
**Diseases of the circulatory system**	**VA-04**	**80**	**17.9**	
Celebrovascular disease	VA-04.03	45	10.1	2
Ischemic heart disease	VA-04.02	17	3.8	
Congestive heart failure	VA-04.05	9	2.0	
Hypertensive diseases	VA-04.01	9	2.0	
**Neoplasm**	**VA-02**	**28**	**6.3**	
Malignant neoplasm of esophagus	VA-02.02	9	2.0	
**External causes**	**VA-11**	**28**	**6.3**	
Accidental falls	VA-11.03	18	4.0	
**Mental and nervous system disorder**	**VA-08**	**22**	**4.9**	
Epilepsy	VA-08.02	10	2.2	
Alzheimer disease	VA-08.01	9	2.0	
**Renal disorder**	**VA-07**	**17**	**3.8**	
Renal failure	VA-07.01	16	3.6	

VA-Verbal Autopsy.

### Broad causes of adult deaths

**Figure 1:**
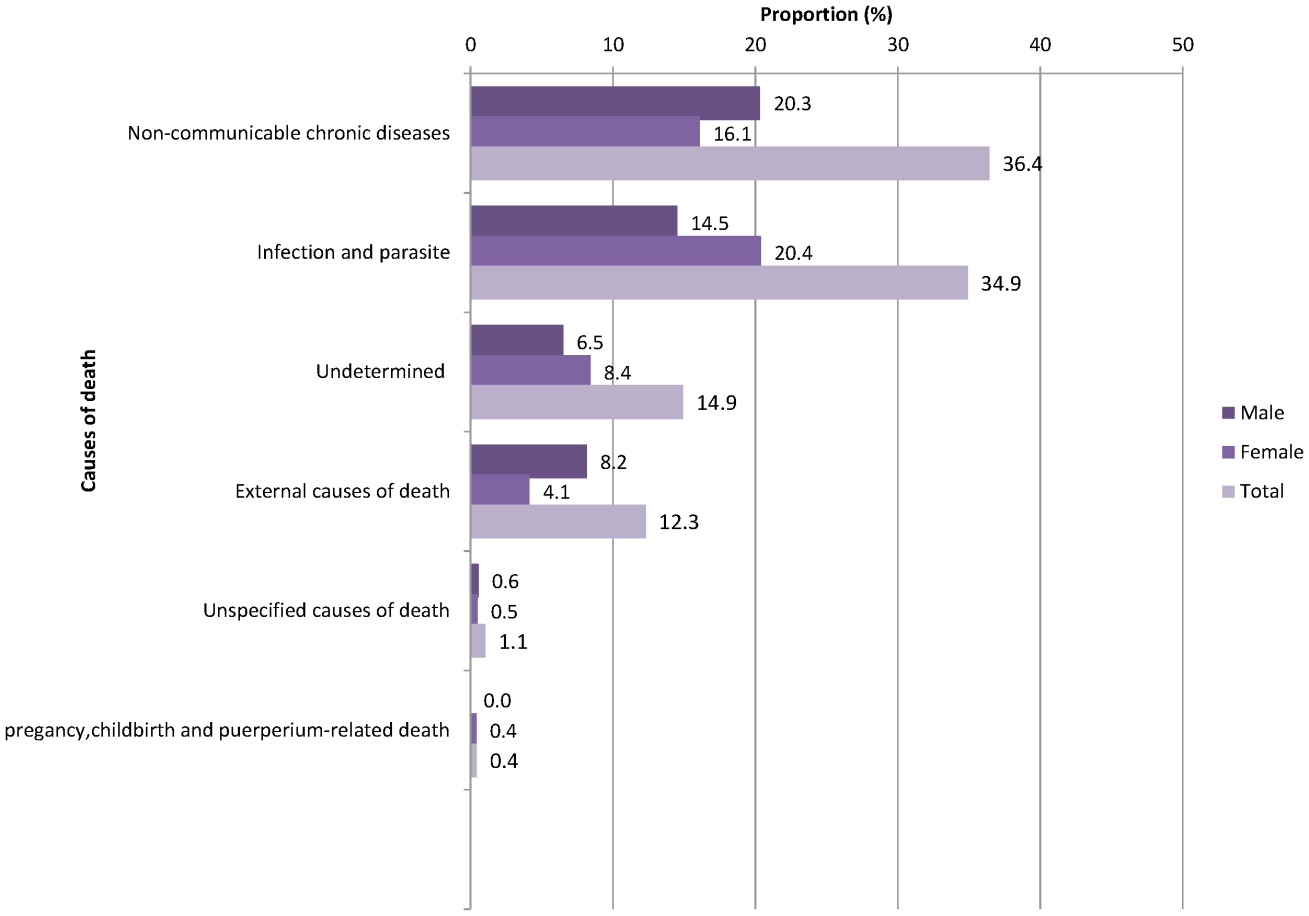
Causes of adult deaths by sex in northern Ethiopia from September 2009 to January 2013 (n = 723).

Among those who had ascribed cause of death (n = 723), 263 (36.4% [95% CI: 32.9, 39.9]) fell within NCDs classification, 252 (34.9% [95% CI: 31.4, 38.4]) were classified within CDs, and 89 (12.3% [95% CI: 10.1, 14.9]) were in the classification of ECs **(** **Figure 1** **)**.

Although a higher proportion of deaths due to NCDs were in males (40.6% [95% CI: 35.6, 45.7]) than females (32.1% [95% CI: 27.5, 37.1]), these proportions were not significantly different. The Cause Specific Mortality Fraction (CSMF) of CDs among females (40.7% [95% CI: 35.7, 45.9]) were significantly higher than their male counterparts (29.0% [95% CI: 24.5, 33.8]). In case of ECs of death, the reverse was true–CSMF was higher among males (16.3% [95% CI: 12.8, 20.4]) compared to females (8.3% [95% CI: 5.8, 11.8]) **(data not shown)**.

**Figure 2:**
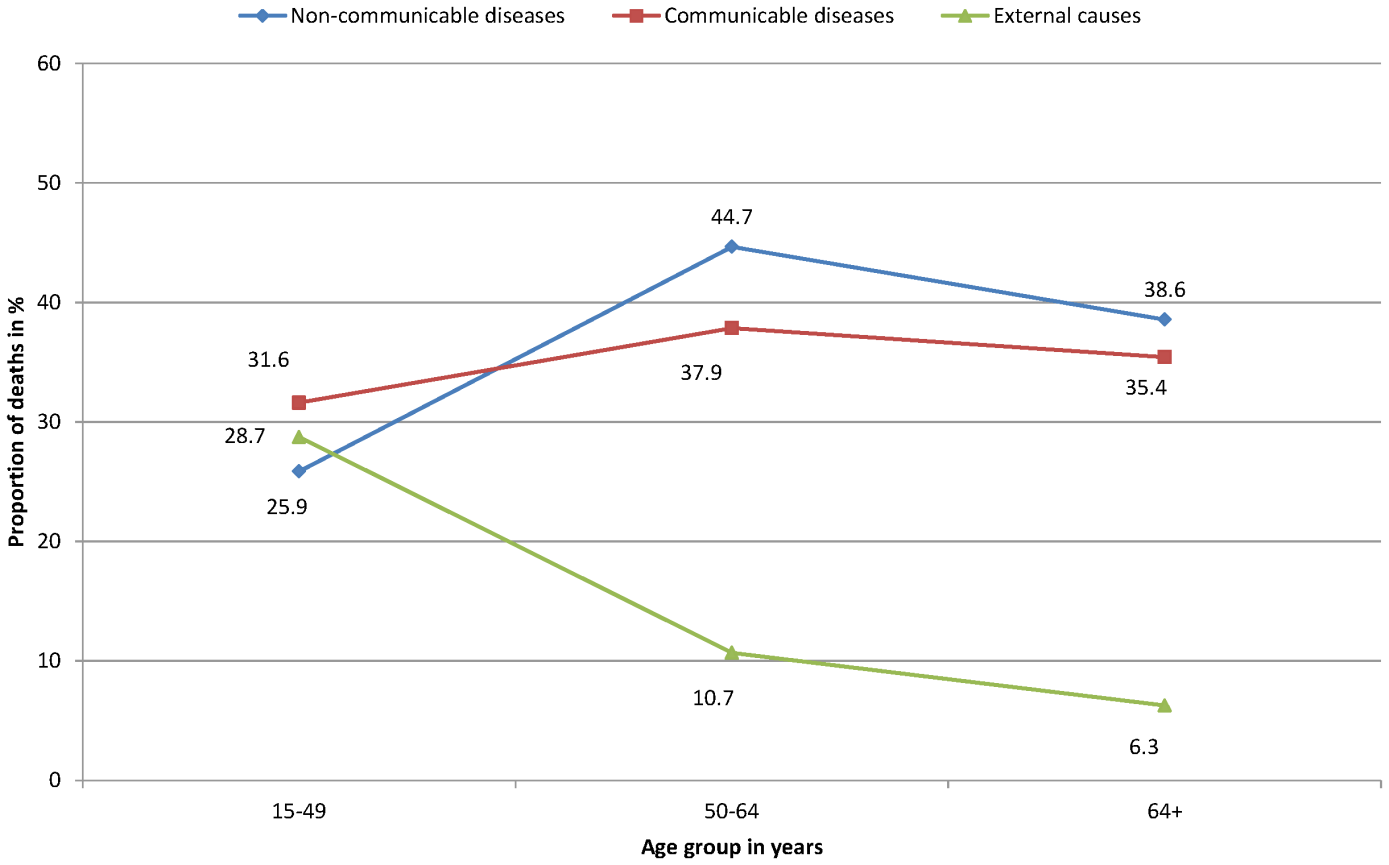
Broad causes of mortality summarized by age group among adults in northern Ethiopia from September 2009 to January 2013 (n = 723). (Communicable diseases, χ^2^ test, p<0.001; non-communicable diseases, χ^2^ test, p<0.001; external causes, χ^2^ test, p<0.001).

The trends in cause of death by age (15–49, 50–64 and 65 years and above) are shown in **Figure 2**. Of all causes in 15–49 years of age, CDs were the leading causes of death accounting for 31.6% of all deaths in the age group. Across all age categories, no significant differences in deaths attributed to CDs were found. NCDs showed an increasing trend as age increased (p<0.001). In contrast, ECs decreased significantly as age increased (p<0.001) **(** **Figure 2** **).**

**Figure 3:**
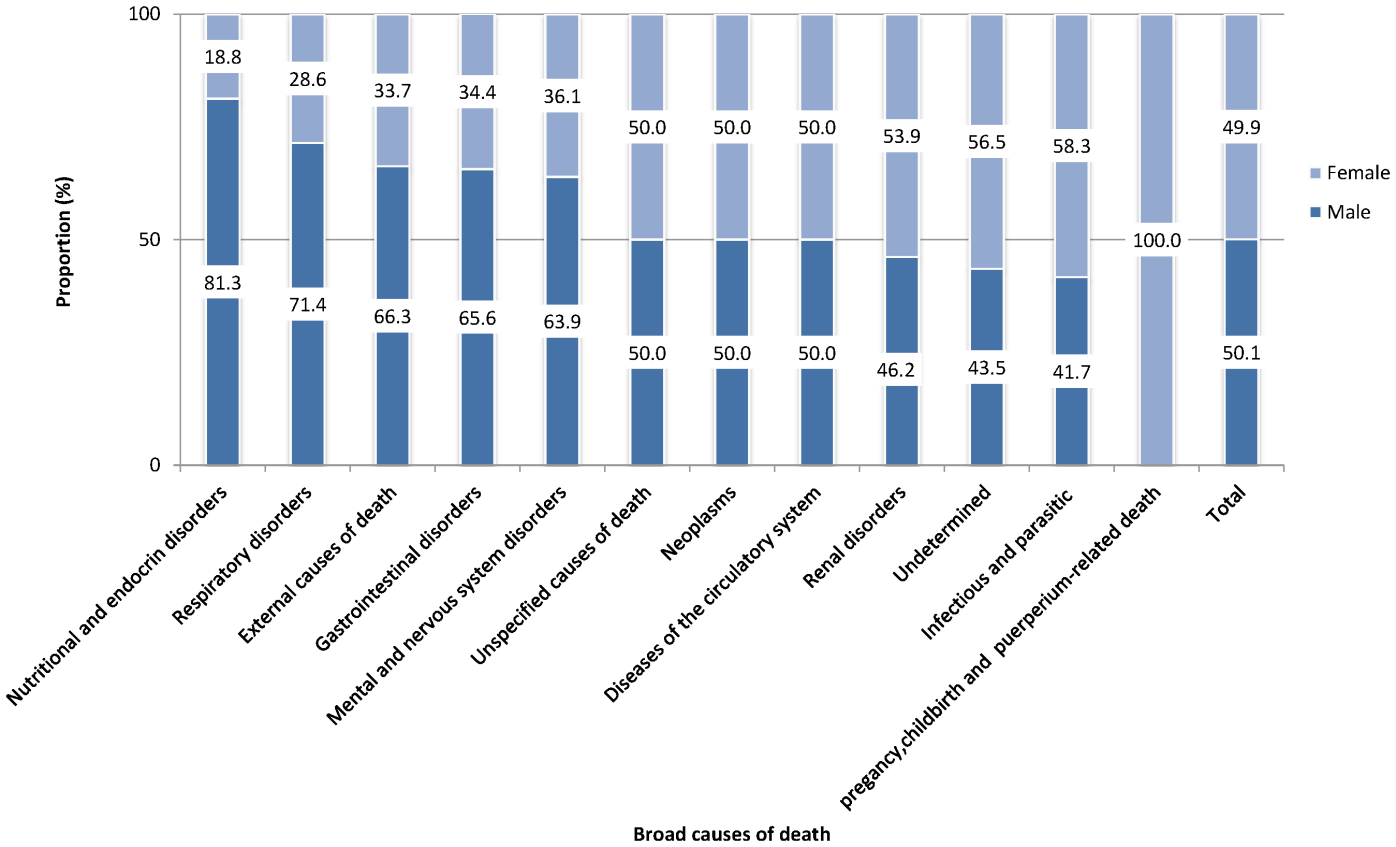
Causes of adult deaths as a proportion of sex in northern Ethiopia from September 2009 to January 2013 (n = 723).

Of all deaths caused by nutrition and endocrine disorders, respiratory disorders, external causes, gastrointestinal, and mental and nervous system disorders, 81.3%, 71.4%, 66.3%, 65.6% and 63.9% occurred among males, respectively. Similar proportions of deaths due to neoplasm and diseases of the circulatory system in both sexes were observed **(** **Figure 3** **)**.

### Association of socio-demographic variables with communicable, non-communicable and external causes of death

In the logistic regression model, sex, age, marital status, occupation and residence were considered. In bivariate logistic regression, sex and marital status were significantly associated with CDs, NCDs and ECs. Age was also associated with NCDs and ECs of death.

In multivariate analysis, females had 1.5 times (AOR = 1.53 [95% CI: 1.10, 2.15]) higher odds of dying due to CDs compared to males. Similarly, if marital status was dissolved (widowed and divorced or separated), a 1.7 times (AOR = 1.73[95% CI: 1.03, 2.90]) higher odds of dying due to CDs was found compared to those never married. The odds of dying due to NCDs significantly increased as the age of person was greater than 50 years of age compared to those in the age group of 15–49 years (**Table 4**).

**Table 4:** Factors associated with communicable diseases, non-communicable diseases and external causes of adult deaths in northern Ethiopia, from September 2009 to March 2013 (n = 723).

		Communicable diseases				
Characteristics	Categories	Yes	No	COR(95% CI)	AOR(95% CI)	P-value
Sex	Male	105	257	1.00	1.00	
	Female	147	214	1.68(1.23,2.29)	1.53(1.10,2.15)	0.013
Marital status	Never married	26	77	1.00	1.00	
	Married	100	213	1.39(0.84,2.30)	1.41(0.85,2.34)	0.181
	Dissolved	120	176	2.02(1.22,3.33)	1.73(1.03,2.90)	0.037
	Unknown	6	5	3.55(1.00,12.62)	3.59(1.00,12.84)	0.049
		Non-communicable diseases				
		Yes	No			
**Sex**	Male	147	215	1.44(1.07,1.96)	1.39(0.96,2.00)	0.078
	Female	116	245	1.00	1.00	
**Age category in years**	15–49	45	129	1.00	1.00	
	50–64	46	57	2.31(1.38,3.87)	**2.15(1.24,3.74)**	**0.007**
	65 and above	172	274	1.80(1.22,2.66)	**1.61(1.04,2.51)**	**0.034**
**Marital status**	Never married	26	77	1.00	1.00	
	Married	128	185	2.05(1.24,3.37)	1.41(0.80,2.49)	0.231
	Dissolved	104	192	1.60(0.97,2.66)	1.28(0.71,2.32)	0.411
	Unknown	5	6	2.47(0.69,8.76)	1.78(0.48,6.58)	0.388
**Occupation**	Farmer	138	190	1.57(1.16,2.13)	1.19(0.83,1.71)	0.334
	Other	125	270	1.00	1.00	
		**External causes**				
		**Yes**	**No**			
**Age category in years**	15–49	50	124	6.02(3.64,9.97)	**4.02(2.25,7.18)**	**<0.001**
	50–64	11	92	1.79(0.86,3.72)	1.72(0.82,3.65)	0.154
	65 and above	28	418	1.00	1.00	
**Sex**	Male	59	303	2.15(1.35,3.42)	**1.70 (1.01,2.85)**	**0.044**
	Female	30	331	1.00	1.00	
**Marital status**	Never married	33	70	8.25(4.30,15.83)	**3.03(1.40,6.56)**	**0.005**
	Married	40	273	2.56(1.40,4.69)	1.69(0.89,3.23)	0.112
	Dissolved	16	280	1.00	1.00	
	Unknown	0	11	N/A	N/A	

COD-Crude Odds Ratio, AOR-Adjusted Odds Ratio.

The odds of dying due to ECs were higher among 15–49 years of age (AOR = 4.02 [95% CI: 2.25, 7.18]) compared to 65 and above years. Males had 1.7 times (AOR = 1.70 [95% CI: 1.01, 2.85]) higher odds of dying due to ECs compared to females. Never married individuals had 3 times (AOR = 3.03[95%CI: 1.40, 6.56]) higher odds of dying due to ECs compared to those people who had a dissolved marital status (**Table 4**).

## Discussion

In this study, NCDs (36.4%) were the leading causes of death followed by CDs (34.8%) and ECs of death (12.3%). Tuberculosis, cerebrovascular diseases and accidental falls were the leading specific causes of death accounting 15.9%, 7.3% and 3.9% of all deaths, respectively.

In our study, more than a third of deaths were due to NCDs which was consistent with previous studies in Ethiopia [32], [33]. Similarly, the finding is in line with the 2006 estimates for low and middle income countries by the Global Burden of Diseases (54%) and World Bank estimates for Madagascar (40%) [29], [34]. The findings of this study are also similar to a Gambian study where NCDs were reported as leading causes of death among adults [35]. Several studies from Ethiopia also reported that NCDs are increasing health problems [32], [36], [37]. The high level of NCDs in the rural setting is likely to be explained by rapid socio-economic development, a larger scale investment in healthcare [38], [39] and an increased life expectancy [38]. In the current study, we also found a high median age (70 years) at death. A survey in rural south western Ethiopia showed that 80% the population surveyed had at least one risk factor for NCDs [37]. NCDs are thought to be common among urban population. Despite this, they were also important causes of death in rural population having no significant lifestyle change. Due to its growing nature, NCDs need to be a focus of prevention and control strategy, as the attention to this has been less in Ethiopia [39], [40].

CDs were the second leading causes of death (34.9%) among adults, age greater than 15 years, which was similar to other studies in Ethiopia and other parts of the world [29], [32], [34], [35], [36]. Yet, the contribution of CDs to the overall deaths in our study was lower than the 47% reported from north western Ethiopia [33] and much lower than the 58% found in Kenya [41]. Despite the variation in the estimates, studies have shown that the burden of CDs has declined in Ethiopia [32], [33]. This could be explained by the improvements in health and socio-economic status of the population. High coverage of primary health care service which reached 92% of the population [6] is among the factors. The national health care program, which focuses on health promotion and prevention of CDs has also played a significant role [39], [40].

The odds of dying due to CDs among females were 1.5 times higher than in males. This finding is similar with other studies in Ethiopia [32], [42]. Generally, studies reported that the proportion of deaths due to CDs decreased with age [32], [34], [43] which is also consistent with our finding. Similarly, if marital status is dissolved, there was 1.7 times higher odds of dying by CDs compared to singles. A possible explanation is that widowed people are more likely to contract CDs as their husbands or wives likely dead of CDs. For example, if the spouse died due to CDs, like HIV/AIDS or tuberculosis, the other spouse is more likely to acquire and die of the same disease.

Examining on specific causes of death, in our study, tuberculosis was the leading cause of death accounting for 15.9% of all deaths. The finding is also in line with studies in sub-Sahara African countries and South Africa [21], [34]. This magnitude of mortality from tuberculosis could be related to high incidence and low detection rate [40]. Despite the extensive expansion of the Direct Observed Treatments (DOTS) service in Ethiopia [44], the global report by WHO in 2011 ranked Ethiopia as 7^th^ among the tuberculosis high burden countries in the world, with an estimated incidence of all forms of tuberculosis of 261 new cases/100,000 pop/year and a case detection rate of smear positive tuberculosis of 72% [45].

ECs were responsible for 12.3% of the deaths. Due to the mountainous and rocky topography of the study area, accidental falls was the leading cause of death accounting for 31.5% of all ECs. The odds of dying due to ECs were 4 times higher in the 15–49 years of age group compared to 65 years and above. Similarly, males had 1.7 times higher odds of dying due to EC compared to females. The findings of our study are very consistent with other studies [32], [33], [34], [41], [43], [46], [47]. The sex difference of deaths from ECs can be attributed to males engaging in more hazardous and risky activities (e.g. employment) than females. Young people are also more vulnerable to ECs as they are less able to predict and prevent injuries from occurring than adults [46].

It is important to acknowledge the limitations of this study, primarily with regard to the high proportion of cases with undetermined causes that were reported for 14.9% of the deaths. Unable to trace and report signs and symptoms of deceased individual correctly prior to death by the interviewee and challenges with physicians agreeing on the probable cause of death on already collected data were factors for high rate of undetermined causes of death. Despite efforts made to improve the quality of data, like continuous training of data collectors and reviewer physicians, supportive supervisions and selecting appropriate respondents, the validity of cause of death ascertained using VA could be affected by different factors like, design and content of questionnaire, timing of interview, skill of interviewers, respondents identified and approach used to derive probable cause of death from VA data [5], [48].

In conclusion, tuberculosis, cerebrovascular diseases, accidental falls, intestinal infectious diseases and infectious diseases (unspecified causes) were the top five causes of death among adults in our study site in northern Ethiopia. Broad causes of death have shown variation across varying age categories. Overall, NCDs were the leading causes of death among adults in KA-HDSS. Deaths due to NCDs were highest among adults between 50–64 years of age. ECs decreased when age increased. Males were more likely to die due to ECs compared to females, and females had a higher risk of death from CDs compared to males. Despite the introduction and implementation of the DOTS strategy, tuberculosis was found to be a leading cause of death. Moreover, a growing burden of NCDs and ECs was observed in the KA-HDSS. Thus, follow-up and social support for tuberculosis and an urge to include NCDs and ECs in the agenda of the Ethiopian health sector are recommended.
